# Cases Illustrative of the Good Effects of Nitrate of Silver in Various Diseases

**Published:** 1819-03

**Authors:** Wm. Balfour


*For the London Medical and Physical Journal.*

### *Cases illustrative of the good Effects of Nitrate of Silver in various Diseases;* by Wm. Balfour, M.D.

WM. BROWNLIE, aged 61, late a serjeant in the 21st or Royal Scotch Fusiliers, struck his right shin against some piece of furniture, when in a state of intoxication, on St. Patrick's day, 1802, in the Island of Barbadoes. All the efforts of the surgeons to heal the wound proved unavailing, not only while the regiment remained in the West-Indies, but after its return to Europe; and Brownlie was discharged, at the age of 44, on two shillings a-day. This man came under my care on the 12th of May last. The ulcer, which had remained open all this while, now extended from the inside of the middle of the tibia, obliquely downwards and outwards, the length of six inches, and about two in breadth. The edges were extremely thick in some places, and jagged in others; the discharge was copious, of a reddish-brown colour, and as thin as water. The whole limb was enormously swelled, from the points of the toes to the knee, with innumerable cracks in the skin, from which a scorbutic sanies oozed, that corroded the neighbouring parts.

I directed the limb to be washed with a solution of aceto-nitrate of lead every day, and the abraded parts to be dressed with ung. hydrar. nitrat. The cavity of the ulcer was filled with soft rags; and a bandage was applied over all, with a considerable degree of tightness, from the points of the toes to the knee. But my hopes of effecting a cure depended solely on the change that nitrate of silver might produce on the quantity and quality of the discharge; I therefore prescribed a quarter of a grain of this medicine, in the form of pill, four times a-day. I visited the patient every day; saw the sores dressed, and applied the bandage with my own hands. At first, the discharge was so copious and thin as to transude any quantity of dressings that could be applied: in the course of a fortnight, however, it became as thick as cream, white and bland. I had now no doubt of being able to cure this shocking inveterate ulcer.

In ten weeks, and after the patient had taken fourteen dozen of pills, the wound was closed; nor was the patient's general health, which was uniformly good, in the least affected by it. An open sore, indeed, of whatever kind, must always be prejudicial to health; for, in proportion to the extent of surface and quantum of discharge, absorption must take place of a matter which cannot fail to contaminate the whole mass of fluids. Hence the rapidity with which the disease runs its course, after ulceration has commenced, in consumption; hence it is that no person who has an ulcer exhibits a healthy countenance.*

*Edinburgh; Nov.* 1, 1818.

* We recal the attention of our readers to the instances formerly related by Dr. Balfour of the efficacy of the same valuable remedy, which were transcribed from the Medico-Chirurgical Journal into one of the Numbers of our last volume.—Edit.

